# Improved ethanol production by a xylose-fermenting recombinant yeast strain constructed through a modified genome shuffling method

**DOI:** 10.1186/1754-6834-5-46

**Published:** 2012-07-18

**Authors:** Wei Zhang, Anli Geng

**Affiliations:** 1 School of Life Sciences and Chemical Technology, Ngee Ann Polytechnic, 535 Clementi Road, Singapore, 599489, Singapore

**Keywords:** *S. cerevisiae*, *P. stipitis*, Yeast, Genome shuffling, Xylose, Cellulosic ethanol

## Abstract

**Background:**

Xylose is the second most abundant carbohydrate in the lignocellulosic biomass hydrolysate. The fermentation of xylose is essential for the bioconversion of lignocelluloses to fuels and chemicals. However the wild-type strains of *Saccharomyces cerevisiae* are unable to utilize xylose. Many efforts have been made to construct recombinant yeast strains to enhance xylose fermentation over the past few decades. Xylose fermentation remains challenging due to the complexity of lignocellulosic biomass hydrolysate. In this study, a modified genome shuffling method was developed to improve xylose fermentation by *S. cerevisiae*. Recombinant yeast strains were constructed by recursive DNA shuffling with the recombination of entire genome of *P. stipitis* with that of *S. cerevisiae.*

**Results:**

After two rounds of genome shuffling and screening, one potential recombinant yeast strain ScF2 was obtained. It was able to utilize high concentration of xylose (100 g/L to 250 g/L xylose) and produced ethanol. The recombinant yeast ScF2 produced ethanol more rapidly than the naturally occurring xylose-fermenting yeast, *P. stipitis*, with improved ethanol titre and much more enhanced xylose tolerance.

**Conclusion:**

The modified genome shuffling method developed in this study was more effective and easier to operate than the traditional protoplast-fusion-based method. Recombinant yeast strain ScF2 obtained in this study was a promising candidate for industrial cellulosic ethanol production. In order to further enhance its xylose fermentation performance, ScF2 needs to be additionally improved by metabolic engineering and directed evolution.

## Background

In recent years, there is a growing interest in the utilization of renewable resources for the production of bioethanol, which has been deemed as the cleanest liquid fuel alternative to fossil fuels. Apart from starch crops and sugarcane, lignocellulosic biomass, such as wood waste and agricultural waste, was considered as the most potential feedstock for bioethanol production as it is the most abundant source of sugars and does not compete with the food resource. Xylose is the 2^nd^ most abundant sugar present in lignocellulosic biomass after glucose. The efficient fermentation of xylose is required to develop economically viable processes for the production of bioethanol from lignocellulosic biomass [1]. *Saccharomyces cerevisiae* is regarded as an industrial working horse for ethanol production because it can produce ethanol in high titre using hexose sugars and have high ethanol tolerance. However it cannot ferment xylose [2]. The yeast, *Pichia stipitis*, is one of the best naturally occurring xylose-fermenting yeasts and it can convert xylose to ethanol in high yield. However, it has low ethanol and sugar tolerance. This feature of *P. stipitis* has limited its use as an industrial strain for large-scale bioethanol production from lignocellulosic biomass. The primary desired traits of an industrial strain required for fermenting lignocellulosic hydrolysate are efficient utilization of hexoses and pentoses, fast fermentation rates, high ethanol production, high tolerance to ethanol, sugars and fermentation inhibitors. [3].

While rational metabolic engineering was effective in improving phenotypes of *S. cerevisiae* strains for xylose fermentation [4], it normally involves the constitutive expression of multiple genes followed by necessary mutagenesis and post-evolutionary engineering. It is therefore tedious, labour-intensive and time-consuming. On the other hand, the whole genome engineering approach, such as genome shuffling, offers the advantage of simultaneous changes at different positions throughout the entire genome without the necessity for genome sequence data or network information. It therefore has advanced the field of constructing phenotypes at a more wild space as compared with the rational tools [5]. Considering the complexity of pathway design for rational metabolic engineering, genome shuffling uses recursive genetic recombination analogous to DNA shuffling [6]. This strategy was successfully applied in rapid strain improvement of both prokaryotic and eukaryotic cells [7,8]. However, this method largely depends on the efficiency of the traditional protoplast fusion techniques, which have the disadvantages of fusant instability, low fusion efficiency, and time-consuming fusant regeneration [9]. The aim of this study is therefore to rapidly construct a recombinant yeast strain with enhanced xylose-fermentation using a modified genome shuffling method. This involves the recursive recombination of the *P. stipitis* genome with that of *S. cerevisiae* through direct genome isolation and transformation. The improved method shares the same advantages with the protoplast fusion-based genome shuffling method for rapid complex phenotype improvement. In addition, it is time-saving, easier to operate and has higher gene recombination efficiency.

## Results

### Modified method of genome shuffling

Protoplast fusion has been regarded as a traditional and effective way to accelerate strain evolution and been applied in many studies. However, it suffers from the disadvantages of low efficiency of fusion induced by polyethylene glycol (PEG), labour-intensive and time-consuming protoplast preparation and fusant regeneration, and fusant instability. The attempt of this study was to develop a rapid and reliable modified genome shuffling method to construct a recombinant yeast strain with improved performance of xylose fermentation. This method was based on the recombination of the whole genomes from different yeast strains in vivo. Genomic DNA of one parent strain was extracted and it was then transferred into the other parental strain to allow the recombination of the two genomes. Potential recombinant strains with the required features were selected on the properly designed screening plates. Their fermentation performance were then evaluated and compared.

Specifically, in this study, *S. cerevisiae* and *P. stipitis* were used as the parents for recombinant yeast strain construction. In the first round, the whole genome of *P. stipitis* was extracted and transferred into *S. cerevisiae* by electroporation. The recombinant strains were selected on YNBX plates containing 6.7 g/L yeast nitrogen base, 50 g/L xylose and 20 g/L agar. Such plates were incubated at 30°C for 7–10 days. *S. cerevisiae* cannot grow under the same conditions [10]. Eight hybrid yeast strains were obtained and they were further evaluated for ethanol production in YNB broth containing 6.7 g/L YNB, 150 g/L xylose, and 50 mM phosphate buffer at pH 7.0 and 30°C for 72 h. The potential recombinant strain with the best ethanol production performance was F1-8 (Table 1). This strain was then used as the starting strain for the second round genome shuffling. 

**Table 1:** Fermentation performance of first round hybrid yeasts in YNBX broth containing 150 g/L xylose

	***P. stipitis***	**F1-1**	**F1-2**	**F1-3**	**F1-4**	**F1-5**	**F1-6**	**F1-7**	**F1-8**
Ethanol yield	0.27±	0.28±	0.29±	0.28±	0.29±	0.29±	0.29±	0.29±	0.31±
(g/g)	0.01	0.01	0.03	0.02	0.02	0.01	0.01	0.02	0.03
Ethanol productivity	0.32±	0.33±	0.35±	0.34±	0.35±	0.36±	0.35±	0.36±	0.38±
(g/L/h)	0.01	0.01	0.01	0.01	0.04	0.02	0.01	0.01	0.02

**Figure 1:**
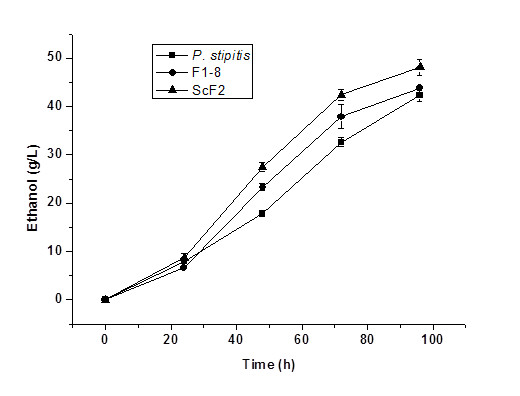
**Fermentation profile of*P. stipitis*, F1-8 and ScF2 in fermentation medium containing 120 g/L xylose.**

In the second round, the whole genome of *S. cerevisiae* was transferred into F1-8 by electroporation and the recombinant strain was screened on YNBXE plates containing 6.7 g/L yeast nitrogen base, 50 g/L xylose, 50 g/L ethanol and 20 g/L agar. Hybrid yeast strain F1-8 showed no growth on this selective plate. Three positive colonies were obtained and the most potential strain was ScF2 according to their competency in ethanol production. As a reference, protoplast fusion was conducted to construct the hybrid yeast using F1-8 and *S. cerevisiae*. None fusants survived on the same YNBXE selective plates. Afterwards, the xylose fermentation capability of the potential recombinant strains F1-8 and ScF2, and their parents, *P. stipitis*, were evaluated in 150 mL shaking flasks filled with 50 mL of the fermentation medium containing 120 g/L xylose. Results are listed in Figure 1. As can be seen, ScF2 presented improved ethanol production rate and ethanol titre compared to both *P. stipitis* and F1-8.

### Random amplified polymorphic DNA (RAPD)

**Figure 2:**
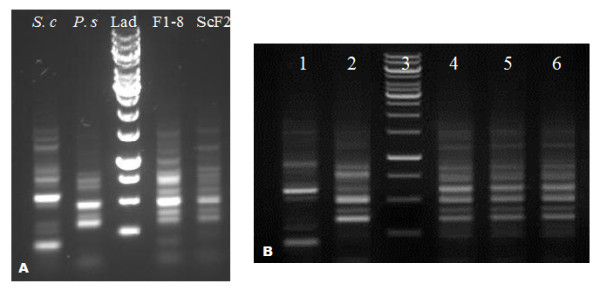
**Genetic variation of yeasts by Random Amplified Polymorphic DNA (RAPD) analysis.A**: RAPD profiles of *S. cerevisiae*, *P. stipitis*, F1-8 and ScF2; Lad is the DNA ladder. **B**: Genetic stability of ScF2; Lanes 1–6 are *S. cerevisiae*, *P. stipitis*, 1 kb DNA ladder, ScF2 (Feb 2011), ScF2 (Jun 2011), and ScF2 (Nov 2011), respectively.

To obtain molecular evidence of the occurrence of recombinatory events using the modified genome shuffling method, we compared the amplification profiles of parental strains and the potential recombinant strains by random amplified polymorphic DNA analysis (RAPD). Using OPA kit, RP1-4, RP-2, RP4-2 and SOY as primers (Table 2), a large number of DNA bands were obtained from the templates of the recombinant yeast strain genomes (Figure 2). Differences were clearly observed between the RAPD profiles of the parents and ScF2 (Figure 2A). Consistent RAPD profiles were obtained for ScF2 obtained at different time point over a period of nine months (Figure 2B).

**Table 2:** Primers used for random amplified polymorphic DNA

**Primers-Operon/Design**	**10-mer in length - 5’ to 3’**
OPA01	CAGGCCCTTC
OPA02	TGCCGCGCTG
OPA03	AGTCAGCCAC
OPA07	GAAACGGGTG
OPA08	GTGACGTAGG
OPA09	GGGTAACGCC
OPA10	GTGATCGCAG
RP1-4	TAGGATCAGA
RP2	AAGGATCAGA
RP4-2	CACATGCTTC
SOY	AGGTCACTGA

### Sugar utilization

The hybrid nature of ScF2 was confirmed by comparing its sugar utilization pattern with those of its two parental strains (Table 3). Combined sugar utilization characteristics of *S. cerevisiae* and *P. stipitis* were observed for the recombinant strain ScF2. ScF2 demonstrated enhanced performance for fructose, xylose, maltose and cellobiose compared to both of the parental strains. It displayed decreased glucose and raffinose utilization capability than *S. cerevisiae*, and less mannose, sucrose and lactose utilization than *P. stipitis*. It showed similar sugar utilization pattern with *P. stipitis* for the rest sugars listed in Table 3.

**Table 3:** Sugar utilization by ScF2 and its parental strains

	***S. cerevisiae***	**ScF2**	***P. stipitis***
control	-	-	-
**Hexoses**			
glucose	+++	++	++
fructose	+	+++	++
galactose	±	+	+
raffinose	±	-	-
mannose	+	+	++
rhamnose	±	+	+
**Pentose**			
xylose	-	+++	++
L-arabinose	-	±	±
D-arabinose	-	-	-
ribose	-	±	±
**Disaccharides**			
sucrose	+	+	++
lactose	-	-	±
cellobiose	-	+++	++
Maltose	+	++	+

-, not growth; ±, feeble growth; +, slow growth; ++, moderate growth; +++, fast growth.

### Fermentation performance of ScF2 in high initial xylose concentration

**Figure 3:**
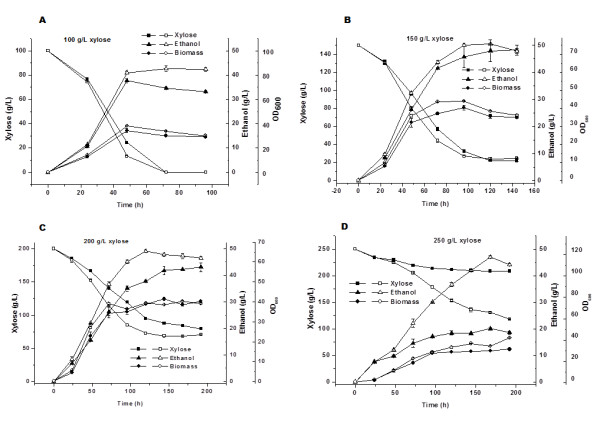
**Time courses of cell growth, xylose consumption and ethanol production by*P. stipitis*and ScF2 in high initial xylose concentration at 30°C and 100 rpm.** Filled symbols, *P. stipitis*; empty symbols, ScF2.

**Figure 4:**
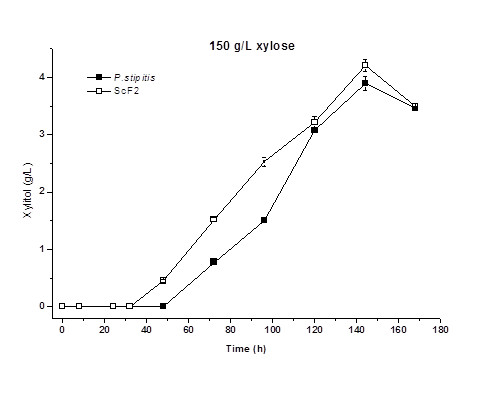
**Time courses of xylitol production by*P. stipitis*and ScF2 in fermentation medium containing 150 g/L xylose.**

In this part of the study, xylose fermentation was conducted in high initial xylose concentration (100, 150, 200, and 250 g/L) using ScF2 and *P. stipitis*. The results are shown in Figure 3. At initial concentration of 100 g/L, xylose was completely utilized on day 3 by both strains and 42 g/L of ethanol was obtained by ScF2 and 38 g/L by *P. stipitis*. The maximum ethanol production of 51 g/L was obtained on day 5 in 150 g/L xylose by ScF2, whereas 48 g/L ethanol was obtained by *P. stipitis* under the same conditions. In addition, recombinant strain ScF2 demonstrated slightly higher rates of xylose consumption and ethanol production in both of the above initial xylose concentration. When the initial xylose concentration was increased further to 200 g/L, the difference between the rates of xylose consumption and ethanol production by ScF2 and *P. stipitis* became more noticeable. Approximately 49 g/L ethanol was obtained by ScF2 on day 5, whereas 43 g/L ethanol was obtained by *P. stipitis* on day 8. At initial xylose concentration of 250 g/L, xylose consumption and ethanol production by *P. stipitis* were significantly inhibited by the high content of xylose and about 20 g/L of ethanol was obtained on day 7. On the other hand, the high xylose content only slightly inhibited xylose consumption and ethanol production by ScF2 with the maximal ethanol concentration of 47 g/L on day 6. The highest ethanol titre of 51 g/L was obtained by the recombinant strain ScF2 in 150 g/L initial xylose concentration. Further increase of the initial xylose concentration triggered a slight decrease in the maximal ethanol titre and an increase of the fermentation time. Although ScF2 demonstrated much higher xylose tolerance and improved ethanol titre compared to *P. stipitis*, its ethanol titre was only limited to around 50 g/L due to the incomplete conversion of xylose. Similar to its parent, *P. stipitis*, the main byproduct for the recombinant strain ScF2 was xylitol. With the enhancement of ethanol production, its xylitol production rate was also higher than that of *P. stipitis* (Figure 4).

### Fermentation of glucose, xylose and their mixture

**Figure 5:**
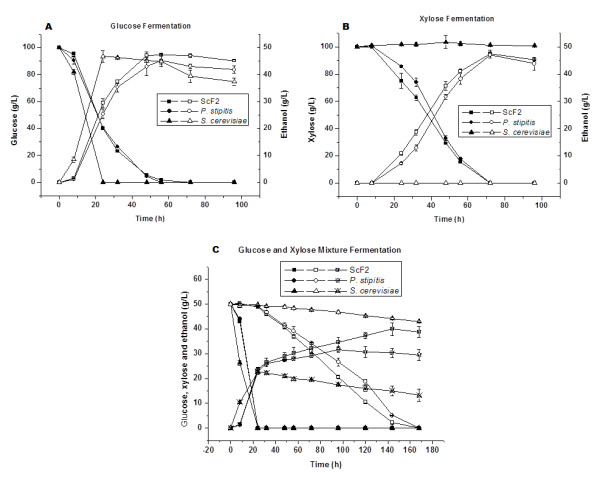
**Time course of glucose, xylose and their mixture fermentation by*P. stipitis, S. cerevisiae*and ScF2.A** and **B**: filled symbols, glucose or xylose; empty symbols, ethanol. **C**: filled symbols, glucose; empty symbols, xylose; crossed empty symbols, ethanol.

In this part of the study, the fermentation of glucose, xylose and their mixture by strains *P. stipitis*, *S. cerevisiae* and ScF2 were investigated independently under batch cultivation conditions. The total sugar concentration was maintained at 100 g/L for all experiments and experiments were conducted in duplicate. As shown in Figure 5, *P. stipitis* and ScF2 could utilize both glucose and xylose, while *S. cerevisiae* could only utilize glucose. Glucose was completely consumed by *S. cerevisiae* within 24 h, by *P. stipitis* within 48 h, and by ScF2 in 56 h. However, ScF2 produced more ethanol (47 g/L) than *P. stipitis* (45 g/L) from glucose. Complete utilization of xylose was observed for both ScF2 and *P. stipitis*, with the former being faster in the rates of both xylose consumption and ethanol production. For the case of glucose and xylose mixture fermentation, again ScF2 and *P. stipitis* could utilize both sugars, with glucose being consumed in a much faster rate. *S. cerevisiae* strain only consumed glucose and the maximal ethanol concentration was 22 g/L. Slight decrease of xylose consumption rate was also observed for both ScF2 and *P. stipitis* under this condition compared to the case when xylose was used as the sole carbon source. In addition, ScF2 exhibited slightly higher rates for both xylose consumption and ethanol production than *P. stipitis*. The maximal ethanol concentration of 40 g/L was obtained for ScF2 at 144 h, and that for *P. stipitis* was 31 g/L at 96 h.

### Xylose fermentation by ScF2 precultured in high-concentration glucose or xylose

**Figure 6:**
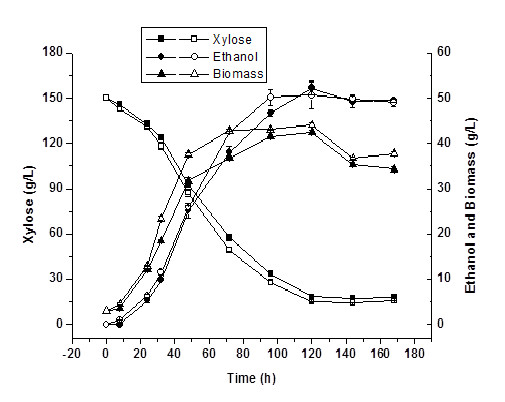
**Fermentation profile of ScF2 precultured in high-concentration glucose or xylose.** Filled symbols, ScF2 precultured on glucose; Empty symbols: ScF2 precultured on xylose.

It was reported that metabolic lag existed for substrate transition [11]. This indicates that yeast strain precultured on glucose prior to its use as inoculum for xylose fermentation may lead to longer metabolic lag phase. In order to further improve xylose fermentation performance by ScF2, seeds culture of ScF2 was prepared in yeast peptone medium containing 10 g/L yeast extract, 20 g/L peptone, and 150 g/L glucose or xylose. Cells were harvested and inoculated to fresh fermentation medium containing 150 g/L xylose at an initial OD_600_ of 3.0. Results are displayed in Figure 6. Slight enhancement of cell growth and ethanol production by ScF2 precultured in xylose were observed. The maximal ethanol titre was obtained at 96 h by xylose precultured ScF2 and at 120 h by glucose precultured ScF2 (Table 4). Interestingly, although preculture in glucose resulted in a slightly longer lag phase for cell growth and ethanol production, a marginally higher ethanol titre, 52 g/L, was obtained compared with the preculture in xylose (Table 4). Noticeably, despite the difference in preculture substrates, ScF2 presented higher xylose consumption rate and ethanol productivity compared to *P. stipitis*. This concurred to the results obtained in previous sessions. 

**Table 4:** Xylose fermentation parameters with ScF2 inoculum pre-cultured in 150 g/L glucose or xylose

		**Ethanol (g/L)**	**Yield (g/g)**	**Xylose (g/L/h)**	**Ethanol (g/L/h)**	**Time (h)**
ScF2	Pre-G	52.25 ± 1.48	0.40 ± 0.01	1.10 ± 0.00	0.44 ± 0.01	120
	Pre-X	50.20 ± 1.78	0.37 ± 0.01	1.28 ± 0.01	0.52 ± 0.02	96
*P stipitis*	Pre-G	52.15 ± 0.28	0.38 ± 0.00	0.95 ± 0.01	0.36 ± 0.00	144
	Pre-X	49.94 ± 0.62	0.37 ± 0.01	0.93 ± 0.01	0.35 ± 0.00	144

## Discussion

*S. cerevisiae* is the best working horse for ethanol industrial production [12]. However, hydrolysate from biomass contains both hexoses and pentoses, and wild-type strains of *S. cerevisiae* cannot utilize pentoses, such as xylose. Utilization of xylose is very important to improve the ethanol yield from biomass hydrolyzate making the process economically viable. Numerous recombinant S*. cerevisiae* strains were constructed by heterologous expression of xylose utilization pathways from *P. stipitis* and overexpression of endogenous XKS gene through rational metabolic engineering in combination with evolutionary engineering [4,13,14]. Potential recombinant strains were obtained with the efforts of scientists from around the world over the past few decades. Protoplast fusion is widely used to improve the fermentative properties of industrial yeasts. It is a potential method to rapidly construct a hybrid strain with combined traits of both parental strains. Attempt of construction the recombinant yeast strain through protoplast fusion of *S. cerevisiae* and *P. stipitis* were made in order to obtain a hybrid yeast with the enhanced ethanol tolerance and xylose fermentation performance [15,16]. Although the hybrid yeast was improved in ethanol tolerance, its xylose fermentation rate and ethanol yield were lower than those of its parent strain *P. stipitis*[16]. In addition, it was discovered that the mononucleate fusants were able to quickly segregate into their parental type strains [17]. More recently, protoplasts of thermotolerant *S. cerevisiae* VS3 and mesophilic, xylose-utilizing *C. shehatae* were fused by electrofusion [3]. The fusants were selected based on their growth at 42 °C and ability to utilize xylose. The mutant fusant CP11 was found to be stable with an ethanol yield of 0.459 ± 0.012 g/g, productivity of 0.67 ± 0.15 g/l/h and fermentation efficiency of 90%. However the maximal ethanol titre obtained was limited to 26–32 g/L.

Genome shuffling uses recursive genetic recombination through protoplast fusion. It is an effective and rapid strategy to obtain a potential strain with improved phenotypes [5]. In this study, we attempted to construct a recombinant yeast strain using a modified genome shuffling method. Instead of using recursive protoplast fusion, recursive direct genome isolation and transformation were used for gene recombination. In the first round, the whole genome of *P. stipitis* was extracted and transferred into *S. cerevisiae*. The recombinant strains were screened on YNBX plates containing 6.7 g/L yeast nitrogen base, 50 g/L xylose, and 20 g/L agar. Eight positive colonies were obtained and they were evaluated for ethanol production in YNB broth containing 150 g/L xylose. One potential recombinant yeast strain F1-8 was selected due to its better xylose fermentation performance (Table 1). This strain was then used as the starting strain for the second round genome shuffling, where the whole genome of *S. cerevisiae* was extracted and transferred into F1-8 and the resulted recombinant strain was screened on YNBXE plates containing 6.7 g/L yeast nitrogen base, 50 g/L xylose, 50 g/L ethanol and 20 g/L agar. Three potential recombinant colonies were obtained and the most potential recombinant strain ScF2 was selected due to its enhanced xylose fermentation performance. The final potential recombinant yeast ScF2 presented improved ethanol production rate and ethanol titre compared to both *P. stipitis* and the first round recombinant strain F1-8 (Figure 1). The results indicate that the modified genome shuffling method adopted in this study is efficient in generating a recombinant yeast strain with improved xylose fermentation capability. In combination with proper screening strategy, this modified genome shuffling was able to rapidly construct a hybrid yeast strain with desired traits from both of the parental yeasts. This modified genome shuffling method was fast, straight-forward, and easy to operate. To our knowledge, this is the first report of such method.

The molecular analysis was carried out to identify the hybrid nature of ScF2. The random amplified polymorphic DNA (RAPD) technique relies on the use of arbitrary primers which are annealed to genomic DNA using low temperature conditions. This technique detects genetic polymorphisms and does not depend on prior knowledge of species-specific sequences [18,19]. From Figure 2A, it can be observed that, apparently, there were differences between the RAPD profiles of ScF2 and its parental strains, suggesting that ScF2 is different from its parents on the genetic level. According to Figure 2A, the RAPD profile of ScF2 was closer to that of *P. stipitis*, indicating that more genetic material in ScF2 might be from *P. stipitis*. Consistent RAPD profiles of ScF2 stored at different time point shown in Figure 2B demonstrate the genetic stability of ScF2. It reconfirmed the high efficiency of gene recombination using the modified genome shuffling method.

Sugar utilization test proved that the potential recombinant yeast ScF2 had the ability to utilize most of the tested pentoses, hexoses and disaccharides (Table 3). Combined sugar utilization characteristics of *S. cerevisiae* and *P. stipitis* were observed for the recombinant strain ScF2 indicating the successful recombination of genomes from both *P. stipitis* and *S. cerevisiae*. Compared to *S. cerevisiae*, ScF2 had better ability to assimilate more sugars and enhanced sugar utilization than *P. stipitis*.

Xylose fermentation performance of ScF2 was tested in fermentation medium initially containing high xylose concentration (100 – 250 g/L xylose). Results displayed in Figure 3 clearly demonstrate that ScF2 exhibited faster rates of both xylose consumption and ethanol production than the naturally occurring xylose fermenting yeast, *P. stipitis*. In addition, it was much more tolerant to the high xylose concentration (Figure 3D) and produced more ethanol under the same cultivation conditions. Such enhancement in rates of ethanol production and sugar tolerance can be attributed to the parent strain *S. cerevisiae*, indicating the recombination of its genes in the hybrid yeast ScF2.

The maximal ethanol production of 51 g/L was obtained on day 5 in fermentation medium initially containing 150 g/L xylose by ScF2, whereas 48 g/L ethanol was obtained on day 8 by *P. stipitis* under the same conditions. Further increase in the initial xylose concentration did not result in further increase of ethanol production. On the contrary, it resulted in the decreased ethanol titre and a longer fermentation time for both ScF2 and *P. stipitis*. It was reported that ethanol plays a dramatic role as a repressor preventing the induction of specific enzymes needed for xylose utilization in *P. stipitis* and when ethanol concentration was greater than 30 g/L, induction of xylose reductase (XR) and xylitol dehydrogenase (XDH) was greatly decreased [11]. Ethanol concentration was topped at around 50 g/L for ScF2 in fermentation medium initially containing increased xylose concentration (100 – 250 g/L), indicating the repression of xylose utilization pathway by ethanol. This feature of ScF2 is similar to that of *P. stipitis* because xylose utilization pathway in both strains was from the same source. However, recombinant *S. cerevisiae* strains constructed by heterologous expression of *P. stipitis* xylose utilization pathway did produce ethanol in a titre higher than 60 g/L [4]. This might be due to the fact that the regulation system in rationally constructed recombinant *S. cerevisiae* strains was from their host, *S. cerevisiae* and these genes were normally expressed using strong constitutive promoters. The limitation of ethanol titre to around 50 g/L by ScF2 indicates that the gene regulation system of the xylose utilization pathway in this hybrid yeast was mainly from *P. stipitis*. Although a titre of 51 g/L ethanol using ScF2 is lower than that using the rationally constructed recombinant *S. cerevisiae*, it is so far the highest ethanol titre obtained by hybrid yeasts. Through traditional protoplast fusion, hybrid yeasts normally presented lower ethanol titre [3,16] and slower ethanol production rates or lower ethanol yield compared to their parents [15,16]. The might be attributed to the instable nature of such hybrid yeast strains due to the different background of the parent species and the limited genetic material transferred through protoplast fusion techniques. Results listed above suggest that the modified genome shuffling method is effective for efficient gene transfer and therefore capable of constructing stable recombinant yeast strains with enhanced fermentation performance in a short time.

It is noticeable that besides ethanol, high xylose concentration was another repressor for *P. stipitis*. With the increase of initial xylose concentration, the difference in rates of xylose consumption and ethanol production between ScF2 and *P. stipitis* became more significant. Higher xylose concentration almost had no effects on the maximal ethanol production for ScF2 (around 50 g/L), though a longer fermentation time was necessary. On the contrary, higher xylose content greatly influenced the maximal ethanol production for *P. stipitis*. When the initial concentration of xylose was increased to 250 g/L, only around 20 g/L of ethanol was obtained by *P. stipitis*. Interestingly, maximal cell biomass growth remained unchanged with the increase of initial xylose content for both ScF2 and *P. stipitis* indicated by the constant OD_600_ at approximately 40, suggesting the inhibition of cell growth under high xylose concentration. Compared to ScF2, higher content of xylose affected more negatively on its rates of xylose consummation and ethanol production for *P. stipitis*, signifying that ScF2 had much better xylose tolerance. The above evidence strongly proves the recombination of *S. cerevisiae* genes in the hybrid yeast ScF2 as *S. cerevisiae* strains are normally more resistant to the osmotic pressure from high sugar concentration [1,12].

As expected, xylitol was the main byproduct for ScF2 (Figure 4) and it was produced in a faster rate in ScF2 with a slightly higher concentration compared to that of *P. stipitis*. It was reported that hybrid yeast constructed through traditional protoplast fusion of *S. cerevisiae* and *P. stipitis*, displayed much more xylitol production [16]. Such results further confirm that the current modified genome shuffling method in combination with proper screening strategy was successful in recombinant yeast strain construction to obtain improved phenotypes from both parents.

The performance of ScF2 was further tested in the fermentation of glucose, xylose and their mixture. Results displayed in Figure 5 demonstrated that ScF2 could utilize both glucose and xylose more rapidly than *P. stipitis* and produced more ethanol. However, the rate of glucose consumption for ScF2 was slower than that for *S. cerevisiae*. Similar to its parent strain *P. stipitis*, in the fermentation of glucose and xylose mixture, ScF2 consumed glucose much faster than xylose. Glucose exhibited repression on xylose consumption for both ScF2 and *P. stipitis*, with effects for the latter being more significant. Compared to *P. stipitis*, ScF2 displayed faster rates of xylose consumption and ethanol production for sugar mixture fermentation and produced more ethanol. Such results are in full agreement with those in previous sessions and further reveal the improved performance of ScF2.

More recently, reports showed that repitched cell populations grown on xylose resulted in faster fermentation rates, particularly on xylose [11]. Sugar transition leads to longer lag phase and using repitched yeasts in the fermented sugar could eliminate the lag phase therefore enhance the fermentation rates. In order to further improve the performance of ScF2, we investigated the effects of seed culture preparation using high-concentration glucose or xylose. Results shown in Figure 6 revealed that seed culture prepared using high-concentration xylose exhibited slightly faster rates of cell growth and ethanol production. However, it did not improve the maximal ethanol concentration (Table 4). Interestingly, seed culture prepared using high-concentration of glucose resulted in higher ethanol production (~52 g/L) for both ScF2 and *P. stipitis*, correspondingly higher ethanol yield. This might be due to the less by-product production under such conditions. Despite the difference in the preculture conditions, ScF2 consistently displayed faster rates for xylose consumption and ethanol production compared to *P. stipitis*. This again confirmed the enhancement of its fermentation performance by the modified genome shuffling method. It is worthwhile noting that the lag phase due to sugar transition in our study was insignificant. This may possibly be attributed to the smaller inoculum size (OD_600_ = 3) used in such experiments compared with what reported in the literature (OD_600_ = 40) [11]. In industrial applications, high inoculum size is not possible. Strain improvement is therefore playing a key role in achieving enhanced fermentation rates and higher ethanol productivity.

From the above analysis, the hybrid yeast ScF2 constructed using the modified genome shuffling method entailed in this study, displayed a higher xylose and ethanol tolerance, presented faster rates of xylose consumption and ethanol production, and produced more ethanol. Combined feature of both parents, *S. cerevisiae* (ethanol and sugar tolerance) and *P. stipitis* (xylose utilization), were evidently shown in ScF2. Furthermore, ethanol repression made the ethanol titre of the hybrid yeast limited to around 50 g/L. However, this ethanol titre for ScF2 was higher than those obtained by hybrid yeasts constructed through traditional protoplast fusion techniques, indicating that the modified genome shuffling method adopted in this study was more efficient in gene transfer and recombination. Through direct genome isolation, genomic DNA was randomly cut to small pieces (> 30 kb). They were then transferred to the host strain through electroporation. This enhanced the gene transfer and recombination efficacy compared to protoplast fusion, for which gene transfer mostly depends on the efficiency of cell fusion. In addition, recursive genome transfer and screening allows further enhancement in gene recombination and sequential addition of the desired traits. Using this method, it is likely to add more desired traits, such as temperature tolerance and inhibitor resistance to the recombinant yeasts to construct a robust yeast strain for cellulosic ethanol industries. Direct fusion of isolated fungal nuclei to yeast protoplast was reported [20]. However, such method involved the preparation of protoplast and the regeneration of fusants. It is therefore tedious and time-consuming. Compared to the protoplast-fusion-based approach, our modified genome shuffling method has advantages of high efficiency, high speed and easy operation. Although the hybrid yeast strain constructed in this study has limited ethanol titre of around 50 g/L, it can be further improved by minimal rational metabolic engineering and directed evolution.

## Conclusion

In this study, we developed a modified genome shuffling method for rapid construction of a recombinant yeast strain from *S. cerevisiae* and *P. stipitis*. In combination with properly designed screening strategy, a potential hybrid yeast ScF2 was constructed. This hybrid yeast displayed improved tolerance to xylose and ethanol, enhanced rates of xylose consumption and ethanol production compared to their parents. Combined with proper screening strategy, the modified genome shuffling method was effective and easy to operate for the construction a recombinant strain with desired phenotypes in a short time. However further strain improvement is possible if such method is integrated with rational metabolic engineering and directed evolution.

## Methods

### Strains and media

Pichia stipitis

 CBS 6054, a haploidy yeast, was obtained from Centraalbureau voor Schimmelcultures (CBS, Baarn) Culture Collection, and it was maintained on YPX agar slants containing (g/L): xylose, 20.0; yeast extract, 10.0; peptone, 20.0; agar, 20.0 at pH 5.5 ± 0.2. *Saccharomyces cerevisiae* ATCC 24860, a diploidy yeast, was procured from American Type Culture Collection (ATCC) and it was maintained on YPD agar slants containing (g/L): glucose, 20.0; yeast extract, 10.0; peptone, 20.0; agar, 20.0 at pH 5.5 ± 0.2. They were stored in YPX or YPD broth containing 20% glycerol at −80°C and were subcultured on YPX and YPD plates, respectively, at regular intervals. Yeast cells from freshly streaked YPD plates were inoculated in YPD broth and incubated at 30°C and 200 rpm for 24 h. Cells were harvested and used as the source for genomic DNA extraction, direct genome transformation or as the inoculum for fermentation experiments.

### Genomic DNA extraction

Cells of *Pichia stipitis* CBS 6054 were cultured in 50-mL centrifuge tubes containing 10 mL YPD broth at 30°C and 200 rpm overnight. They were harvested after centrifugation at 5000 × g at 4°C for 5 min and then were washed with 20 mL sterile water three times. Cells were resuspended in 200 μL lysis buffer (100 mM Tris–HCl pH8.0, 50 mM EDTA and 0.5% SDS) and were transferred to a 1.5 mL microcentrifuge tube. Then 0.2 g glass beads (0.5 mm) were added to resuspend the cells. Cell suspension was thoroughly mixed at the maximal speed on a high speed vortex mixer. After centrifugation at 5000× g for 5 min at 4°C, the supernatant was transferred to a new 1.5 mL microcentrifuge tube and 500 μL phenol:chloroform:isoamyl alcohol (25:24:1) was added to the supernatant. This mixture was then briefly mixed on the vortex mixer and was centrifuged again at 12000 × g and 4°C for 10 min. The upper layer was then withdrawn carefully and was transferred to a new 1.5 mL microcentrifuge tube. One mL ice-cold 95% (v/v) ethanol was added to the supernatant and was briefly mixed by inversion. It was then stored at −20°Cfor 2 h to precipitate the genomic DNA. After that, the sample was centrifuged at 12000 × g and 4°C for 10 min and the supernatant was carefully discarded to retain the genome DNA pellet. Afterwards, 1 mL 75% (v/v) ice-cold ethanol was used to wash the genomic DNA pellet three times and the DNA pellets were then dried by incubation at 37°C for 1 h. The genomic DNA was resuspended in 200 μL of sterile water and was stored −20°C until use.

### Electroporation

The host yeast strain *S. cerevisiae* was cultured in 150-mL shaking flasks containing 50 mL YPD broth at 30°C and 200 rpm overnight. Cells were harvested by centrifugation at 5000 × g and 4°C for 5 min and were washed three times with 20 mL sterile water each time. Cells were resuspended in 20 mL pretreatment-solution (0.1 M lithium acetate, 0.1 M Dithiothreitol (DTT), 0.6 M sorbitol, 0.01 M Tris–HCl of pH7.5) and incubated at room temperature for 30 min. The solution was centrifuged at 5000 × g and 4°C for 5 min and the supernatant was discarded. Cells were then resuspended in 20 mL 1 M sorbitol and centrifuged again under the same conditions. Again, the supernatant was discarded. Cells were then resuspended in 80 μL 1 M sorbitol solution and mixed with 20 μL the isolated *P. stipitis* genomic DNA solution. The mixed solution was transferred into an electroporation cuvette and incubated in ice for about 5 min. Electroporation was then conducted using Gene Pulser Xcell^TM^ electroporation system (Bio-Rad, USA) under the prescribed conditions according to the manufacturer’s instructions. After electroporation, 1 mL 1 M sorbitol solution was added into the cuvette gently. The cuvette was then incubated at 30°C for about 2 h. The transformed cells were then resuspended in 50 mL sterile centrifuge tube containing 5 mL YPD broth and incubated at 30°C and 200 rpm for 3 h. The cultivation broth was spread on the predefined screening plates. Afterwards, the plates were incubated at 30°C for 7–10 days. Positive clones were then selected, subcultured on YPD plates and were evaluated in shaking flasks for xylose fermentation. Potential recombinant strains will be used as the host for next round whole genome transformation.

### Random amplified polymorphic DNA (RAPD)

The RAPD reactions were performed using decamer primers of the OPERON random primer kit (OPA 01, 02, 03, 07, 08, 09 and 10), and the arbitrary primers SOY, RP1-4, RP-2, RP4-2 listed in Table 2[21]. The amplification were conducted with a predenaturation at 94°C for 10 min followed by 44 cycles of thermal denaturation at 94°C (45 sec), primer annealing at 36°C (45 sec), and extension at 72°C (2 min). After that, a 10 min final extension at 72°C was conducted to stabilize the amplified DNA products. Such amplified products were separated by electrophoresis in 1.0% agarose gel, 1 × TAE buffer (40 mM Trisbase-Acetate and 1 mM Na2EDTA, pH8.0) and a constant voltage of 120 V, using a horizontal electrophoresis (Cleaver, UK) followed by staining with SYBR Safe (ABM) and visualization in a UV transilluminator.

### Shaking flask fermentation

One loop of the positive clones was transferred from 1-day YPD plates to 150-mL Erlenmeyer flask containing 50 mL of YPD broth. Yeasts were grown for 24 h at 200 rpm on a rotary shaker at 30°C. A small volume of such seed culture was inoculated to each 150-mL Erlenmeyer flask containing 50 mL of the fermentation medium (FM) containing (g/L) yeast extract, 7; Peptone, 2; (NH_4_)_2_SO_4_, 2; KH_2_PO_4_, 2.05; Na_2_HPO_4_, 0.25 to make an initial inoculum size of 0.5 OD_600_. The Erlenmeyer were shaken at 100 rpm and 30°C. Samples were withdrawn periodically to determine the concentration of sugar, ethanol, xylitol and cell biomass. Fermentation experiments were conducted in duplicate.

### Analytical methods

Cell biomass was monitored spectrophotometrically by measuring absorbance at 600 nm. The measurement was made such that the optical density (OD_600_) of the samples was smaller than 0.70, as obtained by sample dilution. This is to ensure that the Beer–Lambert law applies. Samples were filtered through 0.45 μm filters and stored at −20°C until analysed by a 1200 Series HPLC system (Agilent Technologies Inc.) equipped with a Refractive Index Detector. Sugars, ethanol and xylitol were analysed on a Sugar-PakIcolumn (Waters, USA) at 75°C with the mobile phase of 0.001 mM EDTA-Ca and a flow rate of 0.4 mL/min.

### Sugar utilization tests

Sugar utilization tests were carried out in YNB broth containing 6.7 g/L yeast nitrogen base (YNB) and 2 g/L of various tested sugars individually. ScF2 and its parents (*P. stipitis* and *S. cerevisiae*) were inoculated into 50 mL centrifuge tubes containing 10 mL YNB broth with each tested sugar. YNB broth without sugar was used as the control. These tubes were incubated in an orbital shaker at 200 rpm and 30°C for 48 h and experiments were conducted in duplicate [3]. At the end of the experiments, OD_600_ was measured and compared.

## Abbreviations

ATCC, American Type Culture Collection; CBS, Centraalbureau voor Schimmelcultures; DTT, Dithiothreitol; FM, Fermentation medium; Na_2_EDTA, Disodium ethylenediaminetetra-acetate·2H2O; OD_600_, Optical density at 600 nm; OPA, OPERON random primer; PEG, Polyethylene glycol; RAPD, Random amplified polymorphic DNA; SDS, Sodium dodecyl sulfate; TAE buffer, Tris-acetate and EDTA; XR, Xylose reductase; XDH, Xylitol dehydrogenase; YNB, Yeast nitrogen base; YNBX, Yeast nitrogen base medium supplemented with xylose; YNBXE, Yeast nitrogen base medium supplemented with xylose and ethanol; YPD, Yeast peptone dextrose; YPX, Yeast peptone xylose.

## Competing interests

The authors declare that they have no competing interests.

A patent is pending on this work (Singapore patent application No. 201203879-0).

## Authors’ contributions

Dr. Wei Zhang carried out all the experiments, acquired the data and drafted the manuscript. Dr. Anli Geng participated in the design of the study, performed results interpretation and analysis, and critically revised the manuscript for important intellectual content. All authors read and approved the final manuscript.

## Authors’ information

Dr. Wei Zhang is currently a Research Scientist at School of Life Sciences and Chemical Technology, Ngee Ann Polytechnic, Singapore. He is working on the yeast strain improvement for cellulosic ethanol production.

Dr. Anli Geng is currently the Senior Lecturer and Technology Development Manager at School of Life Sciences and Chemical Technology, Ngee Ann Polytechnic, Singapore. She is also the President of BioEnergy Society of Singapore. Her current research focus is lignocellulosic biomass conversion to fuels and chemicals. Specifically, she is working on microorganism development for the production of industrial enzymes (e.g. cellulase, xylanase and laccase), chemicals (e.g. xylitol, and polyhydroxyalkanoate) and fuels (e.g. ethanol).
